# Sex‐Specific BMI Trajectories in Young People With Type 1 Diabetes: A 20‐Year Retrospective Regional Audit

**DOI:** 10.1155/pedi/4106777

**Published:** 2026-04-06

**Authors:** Connor Oliver-Rose, Lourdes M. Lagera, Aimee Cooper, Paul L. Hofman, José G. B. Derraik, Yvonne C. Anderson

**Affiliations:** ^1^ Department of Paediatrics, Health New Zealand | Te Whatu Ora Taranaki, New Plymouth, New Zealand, waikatodhb.health.nz; ^2^ Department of Paediatrics, Health New Zealand | Te Whatu Ora Waikato, Hamilton, New Zealand, waikatodhb.health.nz; ^3^ Liggins Institute, University of Auckland, Auckland, New Zealand, auckland.ac.nz; ^4^ Starship Children’s Hospital, Health New Zealand | Te Whatu Ora Auckland, Auckland, New Zealand, starship.org.nz; ^5^ Department of Paediatrics: Child and Youth Health, School of Medicine, Faculty of Medical and Health Sciences, University of Auckland, Auckland, New Zealand, auckland.ac.nz; ^6^ Environmental–Occupational Health Sciences and Non-Communicable Diseases Research Centre, Research Institute for Health Sciences, Chiang Mai University, Chiang Mai, Thailand, cmu.ac.th; ^7^ Curtin Medical School, Faculty of Health Sciences, Curtin University, Bentley, Western Australia, Australia, curtin.edu.au; ^8^ The Kids Research Institute, Perth Children’s Hospital, Nedlands, 6009, Western Australia, Australia; ^9^ Child and Adolescent Community Health, Child and Adolescent Health Service, Perth, Western Australia, Australia

**Keywords:** age at diagnosis, body mass index, children, diabetes duration, paediatrics, SDS, sex, weight, z-score

## Abstract

**Background:**

The rates of obesity and type 1 diabetes (T1D) in children and adolescents are increasing in many settings worldwide, but data on weight gain in this group are limited in New Zealand. We examined temporal body mass index (BMI) changes and associated factors in young people with T1D in a mixed urban–rural region.

**Methods:**

This study was a 20‐year retrospective audit of clinical data from a regional paediatric diabetes service (June 2000–March 2021). The primary outcome was BMI standard deviation score (BMI SDS), whose trajectories from diagnosis were examined in association with demographic and clinical factors.

**Results:**

A total of 106 young people with T1D (56% male) were followed for a median of 8.3 years [Q1 = 6.1, Q3 = 11.0; maximum 15.1 years] and attended a median of 21 multidisciplinary clinic visits [Q1 = 12, Q3 = 30; range 1–56 visits]. In females, age at diagnosis modified the association between diabetes duration and BMI SDS (interaction *p* = 0.0009). Girls diagnosed at 4.0 years (age at diagnosis 25^th^ percentile for girls in our cohort) had no statistically significant change in BMI SDS over the first 5 years after diagnosis [−0.13 SDS (95% confidence interval [CI] −0.32 to 0.06); *p* = 0.18], while those diagnosed at 9.5 years (75^th^ percentile) experienced an increase of ≈0.39 SDS (0.08–0.70; *p* = 0.014). Among males, increasing age at diagnosis was associated with lower BMI SDS (*p* < 0.0001), whereas there was no statistically significant association with diabetes duration (*p* = 0.087). At any given duration, boys diagnosed at 11.1 years (age at diagnosis 75^th^ percentile for boys) had a BMI SDS approximately 0.70 lower (95% CI −1.02 to −0.45; *p* < 0.0001) than those diagnosed at 4.7 years (25^th^ percentile).

**Conclusions:**

We observed sex‐specific BMI trajectories in young people with T1D, with age at diagnosis exerting variable influences on BMI SDS trajectory in boys and girls. These findings support individualised T1D management strategies within multidisciplinary care.

## 1. Introduction

Type 1 diabetes (T1D) is a lifelong autoimmune condition and one of the most common chronic diseases in childhood [1, 2]. Approximately 1200 children aged 0–14 years were living with T1D in Aotearoa/New Zealand (henceforth referred to as New Zealand) in 2021 [2]. Some studies suggest that the incidence of T1D has increased over the past decades, especially among older children (10–14 years) and those identifying as European [3], but the data are conflicting [2]. Managing T1D requires lifelong lifestyle changes, including daily insulin administration, blood glucose monitoring and dietary adjustments [1].

Concurrently, childhood obesity rates, including severe obesity, are also increasing worldwide [4, 5]. A 2019 OECD [6] report indicated that New Zealanders aged 5–19 years had the second‐highest combined rate of overweight and obesity (39.5%) among 52 countries, second only to the United States. The 2023/24 NZ Health Survey reported a prevalence of 31.5% for overweight and obesity among children aged 0–14 years [7]. Children identifying as Pasifika and Māori, and those residing in the most socioeconomically deprived areas, were over‐represented in these statistics (58.4%, 38.6% and 41.7%, respectively) [7]. Obesity in children is a public health concern due to its associations with increased risk of physical and psychological comorbidities, including hypertension, dyslipidaemia, type 2 diabetes, metabolic dysfunction‐associated steatotic liver disease, obstructive sleep apnoea, anxiety and eating disorders [8–11]. Moreover, childhood obesity imposes a considerable financial burden due to increased pharmaceutical and healthcare service costs for individuals and society [12].

The obesogenic environment (characterised by overconsumption of energy‐dense, nutrient‐poor foods, pervasive marketing of such products and reduced physical activity) has had a marked impact on obesity prevalence [9]. Consequently, growth trajectories for children and adolescents with T1D may also be affected [13]. Historically, young people with T1D tended to fall in the normal or underweight ranges, and obesity was uncommon [13, 14]. However, the prevalence of overweight and obesity among children with T1D is increasing [13]. One U.S. study reported a sevenfold increase in obesity prevalence among young people with T1D over 18 years, from 3.3% to 22.7% [13]. Nonetheless, detailed data on obesity trends and contributing factors among children and adolescents with T1D remain relatively limited compared to type 2 diabetes [14].

Research on overweight and obesity in young people with T1D is critical, as this combination can magnify cardiometabolic risk and exacerbate mental health issues [10, 14, 15]. Disordered eating behaviours (e.g., excessive dieting and binge eating) and eating disorders (e.g., anorexia nervosa and bulimia nervosa) appear to be more prevalent among adolescents with T1D compared to their peers without diabetes [10, 16]. This is particularly true among individuals with overweight or obesity [17], and is associated with increased frequency of insulin omission, poorer glycaemic control and higher rates of diabetes‐related complications [14, 17, 18]. In this context, we aimed to characterise longitudinal body mass index (BMI) trajectories and associated clinical and demographic factors among children and adolescents with T1D in a mixed urban–rural region of New Zealand.

## 2. Methods

The paediatric diabetes service in Taranaki operates through Health New Zealand | Te Whatu Ora Taranaki. It provides care at both the main facility in New Plymouth (Taranaki Base Hospital) and through satellite services at Hāwera Hospital. These facilities serve communities of approximately 57,000 and 10,000 people, respectively. The regional diabetes service team provides comprehensive care for children and adolescents with diabetes up to 17 years of age, with occasional visits beyond this age during transition to adult services.

We conducted a retrospective audit of clinical records from 1 June 2000 to 31 March 2021. Eligible patients were identified from the paediatric diabetes service records. Data extraction was comprehensive: for each eligible patient, we extracted anthropometric data from all available clinic visits with recorded measurements from the date of T1D diagnosis up to 10 April 2018 (the original audit end‐date), subsequently updating the dataset with additional anthropometric data through 31 March 2021. Anthropometric records prior to mid‐2000 were not available for participants diagnosed before 2000. The standard protocol was 3‐monthly clinic visits; however, resource limitations in 2017 extended appointment intervals to 4 months. The diagnosis of T1D was made as previously described [3].

We extracted clinical and demographic information from patient records, as anthropometric data (height and weight) were routinely measured and recorded over time. During clinics, height was measured to the nearest 1 mm with a Harpenden wall‐mounted stadiometer, and weight to the nearest 0.1 kg using Seca 813 digital scales. BMI was calculated and converted into standard deviation scores (BMI SDS) adjusted for age and sex using the WHO growth standards for younger children [19] and the WHO reference for school‐aged children/adolescents [20]. Overweight was defined as a BMI SDS ≥1.036 to <1.645 (85^th^–94^th^ percentiles) and obesity as ≥1.645 (≥95^th^ percentile) [21].

Demographic data included patient age, biological sex (male or female) and self‐reported ethnicity (based on a hierarchical classification system to assign each patient to a single ethnicity) [22]. Socioeconomic deprivation was estimated with the NZ Index of Multiple Deprivation 2018 (IMD18) [23]. Each participant was assigned an IMD18 decile corresponding to their last recorded residential address closest to the first clinic visit during the audit. The IMD is an overall measure of area‐level deprivation based on ranked Data Zones (small geographical areas encompassing ~712 people) [24]. The IMD deciles range from 1 (least deprived) to 10 (most deprived); for analyses, the cohort was divided into two groups (as close as possible to a 50/50 split), classified as either lower (deciles 1–6) or higher (deciles 7–10) deprivation.

The maximum follow‐up duration was calculated as the time difference between the first and last clinic visits during the audit period. The maximum diabetes duration was defined as the time from T1D diagnosis to the last clinic visit.

### 2.1. Statistical Analyses

Potential associations with BMI SDS were examined using linear mixed models with a repeated‐measures design. Initially, unadjusted models were used to examine associations between BMI SDS and both clinical (age at diagnosis and diabetes duration) and demographic factors (sex, ethnicity [Māori or non‐Māori] and area‐level deprivation [lower vs. higher]). Patient ID was included as a random factor to account for multiple measurements per individual.

A multivariable model was subsequently fitted, including all predictors and testing two‐way interactions. Interaction terms were retained only if statistically significant (*p* < 0.05) or if they improved model fit, as assessed by the Akaike Information Criterion (AIC). In the overall cohort, the final model for BMI SDS included age at diagnosis, diabetes duration, sex, ethnicity, area‐level deprivation and two‐way interactions between age at diagnosis and diabetes duration (*p* = 0.011) and age at diagnosis and sex (*p* = 0.034). Sex‐specific models were refitted due to these sex‐by‐age effects. These models retained all predictors, with the female model additionally including an interaction term (age at diagnosis×diabetes duration). Note that age at visit was not modelled separately because it is inherently defined by age at diagnosis and diabetes duration.

Multicollinearity between predictors was assessed using Pearson correlation coefficients, with |*r*| ≥0.60 as a conservative threshold for potential concern. However, there were no such issues for any of the above‐described analyses. Data were analysed using PROC MIXED in SAS v9.4 (SAS Institute, Cary, NC, USA). Models employed the Kenward–Roger approximation for degrees of freedom and an autoregressive covariance structure. All statistical tests were two‐sided at *p* < 0.05. Data are reported in the text as unadjusted or adjusted β coefficients, or adjusted mean differences, and their respective 95% confidence intervals (CIs).

### 2.2. Ethics

This study was conducted in accordance with the New Zealand Code of Health and Disability Services Consumers’ Rights (Health and Disability Commissioner 1996). While clinical audit studies are exempt from full Health and Disability Ethics Committee review under section 3 of their Standard Operating Procedures v.3.0 (Ministry of Health 2019), the study adhered to all principles outlined in the National Ethical Standards for Health and Disability Research and Quality Improvement 2019 (National Ethics Advisory Committee, Ministry of Health).

## 3. Results

### 3.1. Study Population

During the 20‐year audit, 106 children and adolescents with T1D were cared for by the paediatric services in Taranaki who met the inclusion criteria (Table 1). Patients were diagnosed between 1996 and 2018 at a median age of 7.2 years (range 0.9–15.3 years). A total of 56% were male, and most identified as New Zealand European (81%) or Māori (14%) (Table 1). Patients were followed for a median period of 8.3 years [Q1 = 6.1, Q3 = 11.0 years; maximum 15.1 years] and attended a median of 21 multidisciplinary clinic visits [Q1 = 12, Q3 = 30; range 1–56 visits] where anthropometry was measured. However, the number of clinics per patient varied, as did the range of diabetes duration (Table 2).

**Table 1:** Demographic and clinical data for our study population at the first clinic during the audit, as well as age at diabetes diagnosis.

Variable	Level/summary statistic	Cohort	Males	Females
*n*	—	106	59	47

Sex	Male	59 (56%)	—	—
	Female	47 (44%)	—	—

Ethnicity	NZ European	86 (81%)	48 (81%)	38 (81%)
	Māori	15 (14%)	8 (14%)	7 (15%)
	Other^C^	5 (5%)	3 (5%)	2 (4%)

NZ IMD18^A^	Median [Q1, Q3]	7 [4, 9]	7 [4, 8]	6 [5, 9]
	Lower deprivation	52 (49%)	27 (46%)	25 (53%)
	Higher deprivation	54 (51%)	32 (54%)	22 (47%)

Age at diagnosis (years)	Median [Q1, Q3]	7.2 [4.3, 10.4]	8.6 [4.7, 11.1]	6.6 [4.0, 9.5]
	Range	0.9–15.3	1.2–15.0	0.9–15.3

Age (years)	Median [Q1, Q3]	8.4 [5.3, 11.4]	9.0 [5.4, 11.6]	7.8 [4.8, 11.2]

Diabetes duration (days)	Median [Q1, Q3]	37 [17, 80]	34 [16, 71]	37 [17, 127]

Height SDS	Median [Q1, Q3]	0.44 [−0.23, 1.06]	0.53 [−0.19, 1.43]	0.26 [−0.24, 0.90]

Weight SDS	Median [Q1, Q3]	0.84 [0.21, 1.58]	0.84 [0.06, 1.68]	0.81 [0.29, 1.35]

BMI SDS	Median [Q1, Q3]	0.85 [0.28, 1.36]	0.75 [0.24, 1.27]	0.94 [0.28, 1.49]

BMI status^B^	Below cut‐points	90 (85%)	50 (85%)	40 (85%)
	Overweight	7 (7%)	5 (8%)	2 (4%)
	Obesity	9 (8%)	4 (7%)	5 (11%)

*Note:* Categorical data are presented as *n* (%).

Abbreviations: BMI, body mass index; NZ IMD18, New Zealand Index of Multiple Deprivation 2018; Q1, quartile 1; Q3, quartile 3; SDS, standard deviation score.

^A^The NZ IMD18 is a measure of area‐level deprivation [23]; here, lower deprivation included deciles 1–6 and higher deprivation deciles 7–10, to split the study cohort approximately in half.

^B^Overweight was defined as a BMI SDS ≥1.036 (≥85^th^ percentile for age and sex) to <1.645 (<95^th^ percentile), and obesity as a BMI SDS ≥1.645 (≥95^th^ percentile) based on the WHO growth standards for younger children [19] and the WHO reference for school‐aged children/adolescents [20]; ‘below cut‐points’ are those with BMI SDS <85^th^ percentile.

^C^African, Asian, and other ethnicities were combined to maintain patient confidentiality.

**Table 2:** Data on multidisciplinary team clinic visits, diabetes duration and age among the paediatric patients with type 1 diabetes throughout this 20‐year audit.

Variable	Summary statistic	Cohort	Males	Females
Patients (*n*)	—	106	59	47

Total clinic visits (*n*)	—	2296	1270	1026

Clinic visits per patient (*n*)	Median [Q1, Q3]	21 [12, 30]	21 [12, 29]	21 [9, 32]
	Range	1–56	1–56	1–55

Maximum age (years)	Median [Q1, Q3]	16.1 [13.9, 16.7]	16.1 [14.2, 16.7]	15.9 [11.9, 16.7]
	Range	7.8–18.3	7.8–17.9	9.0–18.3

Maximum diabetes duration (years)	Median [Q1, Q3]	6.9 [5.1, 9.8]	6.4 [4.7, 9.2]	7.9 [5.6, 10.2]
	Range	0.2–15.3	0.2–15.1	1.3–15.3

Maximum follow‐up duration (years)^A^	Median [Q1, Q3]	8.3 [6.1, 11.0]	8.3 [6.7, 10.6]	8.1 [6.1, 11.0]
	Range	0.1–15.1	0.1–15.1	0.1–14.5

*Note*: The data on the minimum age and diabetes duration during the audit are reported as the respective data at the first clinic visit in Table 1.

^A^Defined as the difference between the first and last clinic visits during the audit period.

### 3.2. BMI Trajectories

In unadjusted models, increasing age at diagnosis [β = −0.079 (95% CI −0.114 to −0.044); *p* < 0.0001] was associated with lower BMI SDS. On average, children from less deprived areas had BMI SDS 0.35 lower (95% CI −0.62 to −0.08) than those from more deprived areas [mean BMI SDS 0.68 (95% CI 0.49–0.87) vs. 1.03 (0.84–1.22), respectively; *p* = 0.011]. There were no associations with diabetes duration (*p* = 0.81) or sex (*p* = 0.59).

Among females, age at diagnosis modified the association between diabetes duration and BMI SDS (age×duration interaction *p* = 0.0009), indicating that BMI SDS trajectories over time differed according to age at diagnosis (Figure 1A,C). Within the observed data range (Figure 1C), girls diagnosed later showed steeper increases in BMI SDS with increasing diabetes duration than those diagnosed at younger ages (Figure 1A). Thus, for girls diagnosed at age 4.0 years (age at diagnosis 25^th^ percentile for girls in our cohort), there was no statistically significant change in BMI SDS over the first 5 years since diagnosis [Δ = −0.13 SDS (95% CI −0.32 to 0.06); *p* = 0.18]. In contrast, for those diagnosed at 9.5 years (75^th^ percentile), the model predicted a BMI SDS increase of approximately 0.39 SDS over the first 5 years [equivalent to ≈0.08 SDS per year (95% CI 0.02–0.14); *p* = 0.014].

Figure 1Sex‐specific contour plots and data distributions of BMI SDS by age at type 1 diabetes diagnosis and diabetes duration. (A) and (B) show contour plots of predicted mean BMI SDS (body mass index standard deviation scores) from sex‐specific mixed‐effects models according to age at type 1 diabetes diagnosis (*x*‐axis) and diabetes duration (*y*‐axis) in females and males, respectively. ‘Warmer’ (red) and ‘cooler’ (blue) colours denote higher and lower predicted BMI SDS; contour lines connect points of equal BMI SDS. Mixed‐effects models include repeated measures and are adjusted for age at diagnosis, diabetes duration, ethnicity and area‐level deprivation, with the model for females also including a statistically significant interaction term between age at diagnosis and diabetes duration (*p* = 0.0009). (C) and (D) show the corresponding distributions of observed age at diagnosis and diabetes duration for females (C) and males (D) with one point per clinic visit, illustrating the regions of the covariate space with sparse data. Any estimates in the upper‐right region should therefore be interpreted cautiously, as predictions without empirical data.

**Figure:**
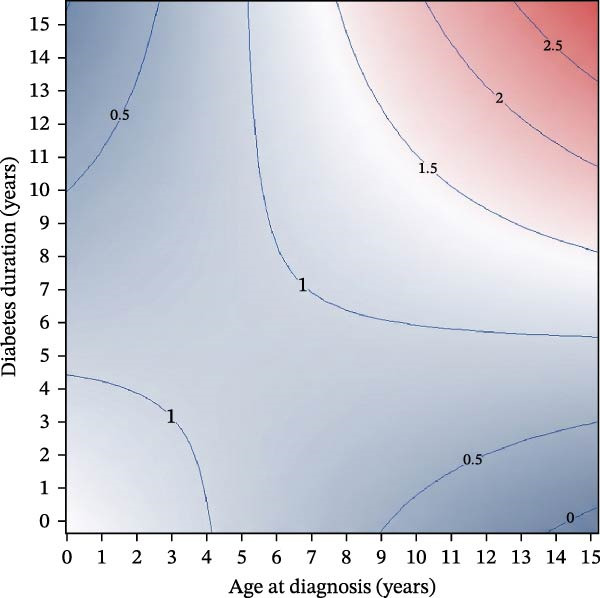
(A)

**Figure:**
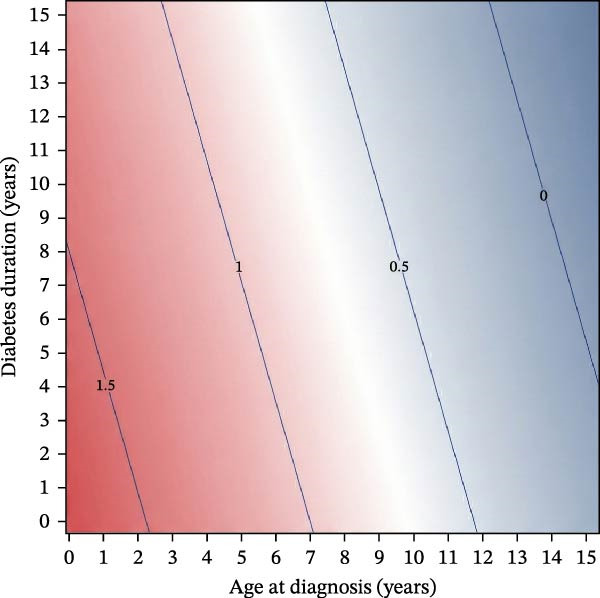
(B)

**Figure:**
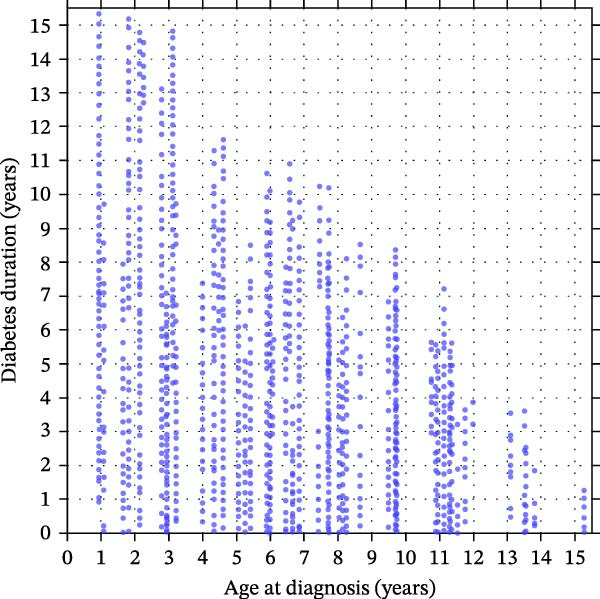
(C)

**Figure:**
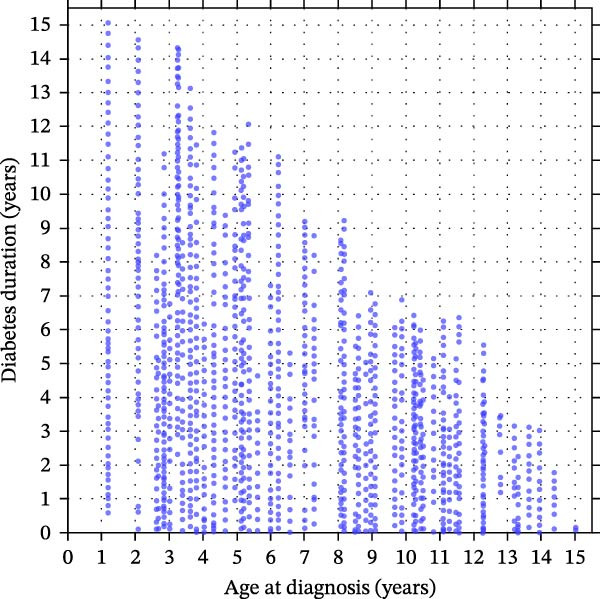
(D)

In males, there was no evidence of an interaction between diabetes duration and age at diagnosis (*p* = 0.66; Figure 1B,D), and diabetes duration was not statistically significantly associated with BMI SDS change [Δ = −0.03 SDS per year (95% CI −0.06 to 0.00); *p* = 0.087]. Conversely, there was a strong association between age at diagnosis and BMI SDS levels (Figure 1B); each additional year of age at diagnosis was associated with a BMI SDS ≈0.11 lower (95% CI −0.16 to −0.07; *p* < 0.0001). For example, at any given diabetes duration, a boy diagnosed at 11.1 years (age at diagnosis 75^th^ percentile for boys) was predicted to have a BMI ≈0.70 SDS lower (95% CI −1.02 to −0.45) than a boy diagnosed at 4.7 years (25^th^ percentile) (Figure 1B).

### 3.3. Other Factors

In the multivariable models, BMI SDS was higher in Māori than in non‐Māori in both females (*p* = 0.045) and males (*p* = 0.043). Notably, there was no evidence that ethnicity modified the associations between age at diagnosis or diabetes duration and BMI SDS over time in either sex. Further, area‐level deprivation was not a statistically significant predictor of BMI SDS among females (*p* = 0.70) or males (*p* = 0.11).

## 4. Discussion

We observed sex‐specific differences in how age at diagnosis and diabetes duration were associated with BMI SDS levels and trajectories in children and adolescents with T1D cared for by a regional service in Taranaki over two decades. These observations align with existing evidence on sex‐specific BMI trajectories in T1D. Studies have shown that female sex, pubertal onset and insulin therapy are important predictors of BMI increase in association with diabetes management [25, 26]. An Israeli study reported an increase in BMI 3–6 years after T1D diagnosis in females but not males [27]. A recent systematic review showed that females with T1D experience a more pronounced increase in BMI, particularly during adolescence, compared to males [25].

The complex interplay between puberty, insulin therapy and metabolic regulation may underpin the observed sex‐specific BMI trajectories. Puberty is typically characterised by increased sex‐steroid secretion and a marked reduction in insulin sensitivity (approximately 25%–30%) [28]. While both sexes undergo these changes, the temporal sequence differs, as girls typically enter puberty 1–2 years earlier than boys [29]. Moreover, the cyclic secretion of oestrogen and progesterone in females has a differential impact on fat mass accumulation and appetite regulation compared to androgens [30], potentially creating a period of heightened susceptibility to weight gain when T1D diagnosis coincides with pubertal onset in girls. These endogenous hormonal changes are further complicated by exogenous insulin therapy, which exerts well‐documented anabolic effects, including enhanced energy storage and reduced catabolism [31]. In addition, the flexibility afforded by intensive regimens may inadvertently contribute to weight gain, for example, via increased caloric intake [32, 33]. Therefore, the convergence of pubertal hormones and therapeutic insulin may explain the accelerated BMI trajectories observed in girls diagnosed near puberty; however, this interpretation requires confirmation with prospective data, including pubertal staging and insulin regimen.

In contrast, we observed that boys diagnosed at younger ages had consistently higher BMI SDS compared to those diagnosed later (e.g., a mean difference of 0.70 SDS between the 25^th^ and 75^th^ percentiles of age at diagnosis). This association between age at diagnosis and BMI SDS appeared relatively stable regardless of diabetes duration, without the progressive increase with diabetes duration seen in girls diagnosed later (around the timing of pubertal onset). The underlying mechanisms require further investigation, as our findings indicate they may be independent of the pubertal hormonal changes that appear to influence BMI trajectories in girls [32, 33]. Early childhood is a critical period for obesity development, when growth and body composition trajectories may be particularly susceptible to environmental and metabolic influences [34]. We speculate that earlier T1D onset may result in exposure to exogenous insulin during these developmentally sensitive periods, potentially contributing to altered metabolic trajectories and increased susceptibility to weight gain. However, diabetes duration itself was not predictive of BMI trajectory in males in our cohort, suggesting that any such effect might be non‐linear, manifest predominantly in the early post‐diagnosis period or reflect metabolic changes established around the time of diagnosis rather than cumulative insulin exposure.

Beyond biological and developmental factors, BMI trajectories in young people with T1D are also affected by their social and environmental contexts [35, 36]. In our cohort, area‐level deprivation was not an independent predictor of BMI SDS after adjustment for other demographic and clinical factors, and did not modify the associations between age at diagnosis or diabetes duration and BMI SDS. This aligns with international evidence noting inconsistent or complex associations between socioeconomic factors and BMI among people with diabetes, contrasting with the clearer gradients typically seen in the general population [36]. However, our findings may also reflect limited statistical power in this regional sample, relatively uniform access to multidisciplinary diabetes care or both. Nonetheless, it remains important to interpret individual BMI trajectories within the wider structural and socioeconomic environment in which families manage T1D [35, 36].

### 4.1. Limitations and Strengths

This study has several limitations. Our study population was relatively small, limiting statistical power and broader generalisability. Moreover, as a retrospective audit of routine clinical data, the timing and completeness of anthropometric measurements varied between patients; however, trained staff used consistent measurement tools and standardised procedures. Further, for each patient, we sought to capture anthropometry from the date of T1D diagnosis, providing a consistent starting point for modelling BMI SDS trajectories and reducing bias due to differences in the availability of early post‐diagnosis data. The exceptions were four patients diagnosed before 2000, for whom anthropometric data were not available prior to 2000; thus, their early post‐diagnosis BMI trajectory could not be ascertained. In addition, ethnicity was self‐prioritised, as was standard hospital practice during the time of this audit [22]. Notably, we had no data on regular physical activity or structured sports participation, which are known to change over time in age‐ and sex‐specific manners [37–39], including in New Zealand [40]. Sex differences in physical activity and sport participation largely reflect sociocultural, psychosocial and environmental factors [38, 40, 41], which could have contributed to the observed sex disparities in BMI trajectories.

Nevertheless, the strengths of our study include the two‐decade audit period in an urban–rural region often under‐represented in clinical research, which provided extensive longitudinal data capturing pre‐ and post‐pubertal stages for most participants. Furthermore, the high frequency of clinical encounters (median: 21 multidisciplinary visits per patient) and substantial follow‐up duration (median: 8.3 years) enabled detailed observation of BMI trajectories following T1D diagnosis across important developmental periods. This comprehensive temporal coverage supports our ability to draw conclusions about sex‐specific BMI patterns in young people with T1D.

## 5. Conclusions

This 20‐year audit showed sex‐specific BMI trajectories among young people with T1D, suggesting distinct patterns in BMI change/weight gain. In females diagnosed later (closer to puberty), BMI SDS increased with longer diabetes duration, whereas this association was absent in those diagnosed earlier. In males, earlier diagnosis was associated with higher BMI SDS, a relationship largely unaffected by diabetes duration. These findings highlight the potential importance of integrating sex‐specific and age‐related considerations into individualised T1D management strategies. Further prospective studies are warranted to elucidate the underlying mechanisms and evaluate targeted interventions for this population. Optimal management is likely to include specialised multidisciplinary team‐based care that carefully balances glycaemic control through insulin therapy with lifestyle interventions to mitigate long‐term metabolic complications [42, 43], while remaining cognisant of potential sex‐specific developmental trajectories.

## Author Contributions

Study design: Connor Oliver‐Rose, José G. B. Derraik, Paul L. Hofman and Yvonne C. Anderson. Data collection: Aimee Cooper and Connor Oliver‐Rose. Data curation: Aimee Cooper, Connor Oliver‐Rose, José G. B. Derraik, Lourdes M. Lagera and Yvonne C. Anderson. Data analyses: José G. B. Derraik. Data interpretation: all authors. Manuscript drafting: Connor Oliver‐Rose, Lourdes M. Lagera, Yvonne C. Anderson, Paul L. Hofman and José G. B. Derraik.

## Funding

Lourdes M. Lagera was funded by the Maureen Lonsdale Summer Studentship, administered by Tamariki Pakari Child Health and Wellbeing Trust. José G. B. Derraik was supported by the Tamariki Pakari Child Health & Wellbeing Trust and Chiang Mai University. Open access publishing facilitated by The University of Auckland, as part of the Wiley—The University of Auckland agreement via the Council of Australasian University Librarians.

## Disclosure

All authors critically revised the manuscript and approved the final submitted version. Following any use of AI, the authors reviewed and edited the content as needed and take responsibility for the manuscript’s content.

## Conflicts of Interest

The authors declare no conflicts of interest.

## Data Availability

Due to the confidential and sensitive nature of the clinical audit data, public access to this dataset is restricted in accordance with the protection of participant health information. Explicit consent to publicly share these data was not obtained during the audit.
